# *SHROOM4* Variants Are Associated With X-Linked Epilepsy With Features of Generalized Seizures or Generalized Discharges

**DOI:** 10.3389/fnmol.2022.862480

**Published:** 2022-05-17

**Authors:** Wen-Jun Bian, Zong-Jun Li, Jie Wang, Sheng Luo, Bing-Mei Li, Liang-Di Gao, Na He, Yong-Hong Yi

**Affiliations:** Institute of Neuroscience and Department of Neurology, Key Laboratory of Neurogenetics and Channelopathies of Guangdong Province and the Ministry of Education of China, The Second Affiliated Hospital of Guangzhou Medical University, Guangzhou, China

**Keywords:** epilepsy, *SHROOM4* gene, whole-exome sequencing, intellectual disability, genotype-phenotype correlation, sub-regional effect

## Abstract

**Objective:**

*SHROOM4* gene encodes an actin-binding proteins, which plays an important role in cytoskeletal architecture, synaptogenesis, and maintaining gamma-aminobutyric acid receptors-mediated inhibition. *SHROOM4* mutations were reported in patients with the Stocco dos Santos type of X-linked syndromic intellectual developmental disorder (SDSX; OMIM# 300434). In this study, we investigated the association between *SHROOM4* and epilepsy.

**Methods:**

Trios-based whole-exome sequencing was performed in a cohort of 320 cases with idiopathic generalized epilepsy or idiopathic partial epilepsy. Protein modeling was used to assess the damaging effects of variations.

**Results:**

Six hemizygous missense *SHROOM4* variants, including c.13C > A/p. Pro5Thr, c.3236C > T/p.Glu1079Ala, c.3581C > T/p.Ser1194Leu, c.4288C > T/p.Arg1430Cys, c.4303G > A/p.Val1435Met, c.4331C > T/p.Pro1444Leu, were identified in six cases with idiopathic epilepsy without intellectual disability. All patients presented with features of generalized seizures or generalized discharges. These hemizygous variants had no or extremely low allele frequencies in controls and showed statistically higher frequency in the case cohort than controls. All variants were predicted to alter hydrogen bond with surrounding amino acids or decreased protein stability. The *SHROOM4* variants reported in patients with SDSX were mostly destructive or duplicative variants; in contrast, the *SHROOM4* variants were all missense variants, suggesting a potential genotype-phenotype correlation. The two missense variants associated with SDSX were located in the middle of SHROOM4 protein, whereas variants associated with idiopathic epilepsy were located around the N-terminal PDZ domain and the C-terminal ASD2 domain.

**Significance:**

*SHROOM4* was potentially a candidate pathogenic gene of idiopathic epilepsy without intellectual disability. The genotype-phenotype correlation and sub-regional effect helps understanding the mechanism underlying phenotypic variation.

## Introduction

*SHROOM4* gene (OMIM* 300579) (also known as *KIAA1202* gene) resides on Xp11.22 and encodes a member of the actin-binding proteins of shroom family (Hagens et al., 2006), which contain a PDZ and an ASD2 domain. The SHROOM4 protein is broadly distributed, including the brain and predominantly during embryonic and adult period. SHROOM4 and members of this protein family have been shown to localize at the cytoskeleton, and play a role in neurulation, cellular architecture, actin remodeling, ion channel function, and synaptogenesis (Hagens et al., 2006; Zapata et al., 2017). *SHROOM4* mutations have been demonstrated to be associated with Stocco dos Santos type of X-linked syndromic intellectual developmental disorder (SDSX; OMIM# 300166) (Froyen et al., 2007; Honda et al., 2010; Armanet et al., 2015; Farwell et al., 2015). Knockdown of *SHROOM4* in rat severely impairs gamma-aminobutyric acid (GABA) receptors activity causing increased anxiety-like behavior and susceptibility to seizures (Zapata et al., 2017). However, the relationship between *SHROOM4* gene mutations and epilepsy is not determined.

In this study, we performed trio-based whole-exome sequencing (WES) in a cohort of patients with idiopathic epilepsy. Six novel hemizygous missense variants of *SHROOM4* were identified in six unrelated cases with epilepsy with generalized seizures or generalized discharges on electroencephalography (EEG). To understand the mechanism of phenotypic variation, we analyzed the genotype-phenotype correlation and the sub-regional effect of *SHROOM4* variants.

## Materials and Methods

### Patients

A total of 320 cases (trios) with epilepsy without acquired causes (idiopathic epilepsies) were recruited in this study from the Epilepsy Center of the Second Affiliated Hospital of Guangzhou Medical University in China between January 2013 and December 2020. The complete pedigree and clinical data of the probands were collected, including age, gender, age of seizure onset, type, course, and frequency of seizure, family history, therapy, prognosis, general and neurological examination, long-term video EEG, and brain magnetic resonance imaging (MRI). Epilepsy syndromes and epileptic seizures were diagnosed according to the criteria of the Commission on Classification and Terminology of the ILAE (1981, 1989, 2001, 2010, and 2017). Idiopathic generalized epilepsy were diagnosed based on a range of seizure types including absence, spasms, myoclonus, clonic, atonic, tonic, and tonic-clonic seizures, supported by typically generalized discharges on EEG. Idiopathic partial epilepsy had partial seizures and features of bilateral discharge or tendency of generalized discharge. EEG examinations showed focal abnormalities with features of idiopathic epilepsies, including shifting, bilateral, or multiple focal discharges with normal backgrounds. Patients with acquired causes were excluded. All subjects were followed up for at least one year.

This project was approved by the Ethics Committee of the Second Affiliated Hospital of Guangzhou Medical University and was conducted according to the guidelines of the International Committee of Medical Journal Editors regarding patients’ consent for research or participation. Written informed consent was obtained from the patients and their legal guardians.

### Whole-Exome Sequencing and Genetic Analysis

Peripheral blood samples were obtained from the probands, parents, and other available family members to determine the origin of the identified genetic variants. Genomic DNA was extracted as previously reported (Wang et al., 2018; Shi et al., 2019). Trio-based Whole-Exome Sequencing (WES) was performed with the Illumina HiSeq 2500/4000 platform by BGI-Shenzhen (Shenzhen, China). Deep sequencing data were aligned to the reference GRCh37 build (hg19) and variants were called according to the standard procedures as previously reported (Shi et al., 2019). A case-by-case analytical approach was used to identify candidate causative variants in each trio. Generally, the rare variants with a minor allele frequency < 0.005 were first prioritized in the 1000 Genomes Projects, Exome Aggregation Consortium, and Genome Aggregation Database (gnomAD) (Genomes Project et al., 2015; Karczewski et al., 2020). Next, potentially pathogenic mutations were retained, including frameshift, nonsense, canonical splice site, initiation codon, and missense mutations predicted as being damaging by *in silico* tools^1^. Finally, potential disease-causing variants were screened under following five models: (1) epilepsy-associated gene model; (2) *de novo* dominant inheritance model; (3) autosomal recessive inheritance model, including compound heterozygous and homozygous variants; (4) X-linked inheritance model; (5) co-segregation model. To identify novel epilepsy-associated genes, the known epilepsy-associated genes (Wang et al., 2017) were not analyzed in the present study. Genes with repetitively identified *de novo* variants, bi-allelic variants, hemizygous variants, or variants with segregations, were selected for further studies to define the gene-disease association. *SHROOM4* appeared as one of the candidate genes with recurrent hemizygous variants in this cohort. All variants in *SHROOM4* were annotated based on transcript NM_020717.5. Positive findings and the variant origination were validated by Sanger sequencing.

### Mutation Analysis

Protein modeling was conducted *via* Iterative Threading ASSEmbly Refinement (I-TASSER) software to predict the effect of candidate variants on molecular structure (Zhang, 2008; Yang and Zhang, 2015). Three-dimensional protein structure and hydrogen bonds alteration were visualized and analyzed by using PyMOL 1.7.

I-Mutant server was used to predict protein stability alteration caused by single nucleotide mutations-related amino acid change (Capriotti et al., 2005). Protein stability was measured with free energy change value (DDG, kcal/mol).

To evaluate the genotype–phenotype correlation, *SHROOM4* variants were systematically reviewed through PubMed database and human gene mutation database up to December 2021.

### Statistical Analysis

IBM SPSS Statistics 19 was used for statistical analysis. Fisher’s exact test was applied to access the allele frequencies of *SHROOM4* variants in the cohort of this study and the control populations, and the proportions of missense variants between epilepsy and intellectual disability. A *p* value of < 0.05 was considered to be statistically significant.

## Results

### Identification of *SHROOM4* Variants

**FIGURE 1:**
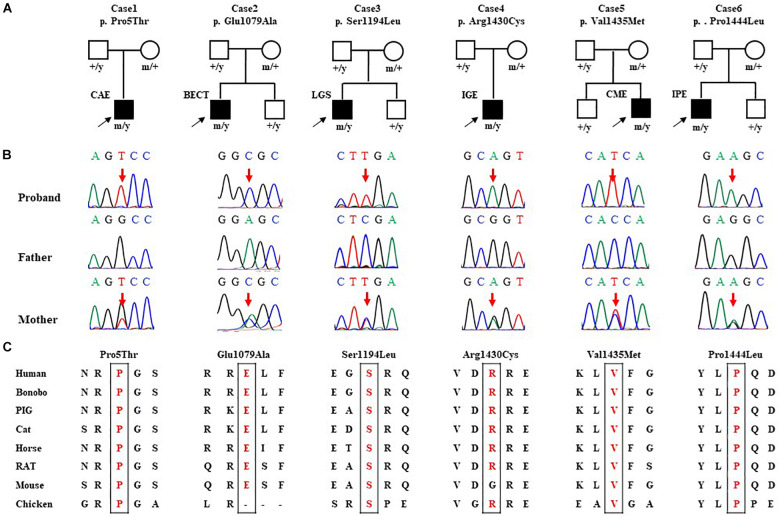
Genetic data of cases with *SHROOM4* variants. **(A)** Pedigrees of the six cases with *SHROOM4* mutations and their corresponding phenotypes. **(B)** DNA sequence chromatogram of the *SHROOM4* mutations. Arrows indicate the positions of the mutations. **(C)** The amino acid sequence alignment of the six missense mutations shows that the residues Pro5, Glu1079, Ser1194, Arg1430, Val1435, and Pro1444 are highly conserved across species.

Six hemizygous missense variants in *SHROOM4*, including c.13C > A/p.Pro5Thr, c.3236C > T/p.Glu1079Ala, c.3581C > T/p.Ser1194Leu, c.4288C > T/p.Arg1430Cys, c.4303G > A/p.Val1435Met, and c.4331C > T/p.Pro1444Leu, were identified in six unrelated cases with idiopathic epilepsy (Figure 1 and Table 1). All of the hemizygous missense variants were inherited from their asymptomatic mothers, consisted with a classical X-linked recessive (XLR) inheritance pattern (Figures 1A,B).

**TABLE 1:** Clinical features of the individuals with *SHROOM4* variants.

Case	Variants (NM_020717.5)	Gender	Age	Seizure onset	Seizure course	Effective AEDs	Seizure-free duration	EEG	Brain MRI	Development	Diagnosis
Case 1	c.13C > A/p.Pro5Thr	Male	9 yr	7 yr	Absence, 4-5 times/day	VPA	1 yr	3 Hz generalized spike-slow waves	Normal	Normal	CAE
Case 2	c.3236A > C/p.Glu1079Ala	Male	11 yr	7 yr	GTCS, 2 times in 2 years	LTG	3 yr	Bilateral central-temporal independent sharp waves and spikes	Normal	Normal	BECTS
Case 3	c.3581C > T/p.Ser1194Leu	Male	16 yr	3 yr	Tonic seizure, 4-5 times/day in the first three years, CPS 1-2 times/month	VPA,OXC,CNZ	2 yr	Childhood: not available Present: generalized slow waves and left temporal spike-slow waves	Normal	Normal	LGS
Case 4	c.4288C > T/p.Arg1430Cys	Male	17 yr	16 yr	GTCS 1-2 times/month	VPA	1 yr	Irregular generalized spike-slow waves	Normal	Normal	IGE
Case 5	c.4303G > A/p.Val1435Met	Male	5 yr	4 yr	Myoclonic seizure, 5-6 times/day	LTG	2 yr	Irregular polyspike-slow waves and bilateral temporal independent spike-slow waves	Normal	Normal	CME
Case 6	c.4331C > T/p.Pro1444Leu	Male	17 yr	3 yr	GTCS or CPS 2-3 times/day	VPA,OXC	1.5 yr	Right predominant generalized spike-slow waves, bilateral temporal independent spike-slow waves Ictal: GTCS with generalized origination	Normal	Normal	IPE

*AEDs, antiepileptic drugs; BECTS, benign childhood epilepsy with centrotemporal EEG spikes; CAE, childhood absence epilepsy; CME: Childhood myoclonic epilepsy; CPS, complex partial seizure; EEG, electroencephalogram; GTCS, generalized tonic-clonic seizure; LGS, Lennox-Gastaut syndrome; LTG, lamotrigine; MRI, magnetic resonance imaging; IGE, idiopathic generalized epilepsy; IPE, idiopathic partial epilepsy; VPA, valproate.*

No hemizygote of these variants were found in the controls of gnomAD-all populations, except the variant Ser1194Leu with an extremely low frequency (1/87,776) (Table 2). All of the hemizygous variants were not found in the controls of gnomAD-East Asian populations.

**TABLE 2:** Analysis of the aggregate frequency of *SHROOM4* variants identified in this study.

Variants (NM_020717.5)	Position	Allele count/number in hemizygotes in this study (%)	Allele count/number in hemizygotes of gnomAD-all populations (%)	Allele Count/number in hemizygotes in controls of gnomAD-all populations (%)	Allele Count/number in hemizygotes of gnomAD-East Asian populations (%)	Allele Count/Number in the controls of gnomAD-East Asian populations (%)
c.13C > A/p.Pro5Thr	chrX:50557006	1/448 (0.2232)	-	-	-	-
c.3236A > C/p.Glu1079Ala	chrX:50350906	1/448 (0.2232)	-	-	-	-
c.3581C > T/p.Ser1194Leu	chrX:50350561	1/448 (0.2232)	1/204,757	1/87,776	-	-
c.4288C > T/p.Arg1430Cys	chrX:50339889	1/448 (0.2232)	1/180,876	-	-	-
c.4303G > A/p.Val1435Met	chrX:50339874	1/448 (0.2232)	-	-	-	-
c.4331C > T/p.Pro1444Leu	chrX:50339846	1/448 (0.2232)	0/181,847	0/79,656	-	-
Total		6/448 (1.3392)	2/180,876	1/79,656	0/13,792	0/6,901
*P* value			6.134 × 10^–15^	4.273 × 10^–13^	9.386 × 10^–10^	9.462 × 10^–8^
OR			1,227.652	1,081.289	405.203	202.756
(95%CI)			(247.11−6,099.01)	(129.91−8,999.96)	(22.72−7,225.75)	(11.37−3,615.63)

*p values and odds ratio were estimated with 2-sided Fisher’s exact test.*

*CI, confidence interval; gnomAD, Genome Aggregation Database; OR, odd ratio.*

The aggregate frequency of the hemizygous variants in this cohort was significantly higher than that in controls (Table 2), including the gnomAD-all population (6/448 vs. 2/180,876; *p* = 6.134 × 10^–15^), the controls of gnomAD-all population (vs. 1/79,656; *p* = 4.273 × 10^–13^), the gnomAD-East Asian population (vs. 0/13,792, p = 9.386 × 10^–10^), and the controls of the gnomAD-East Asian population (vs. 0/6,901, *p* = 9.462 × 10^–8^).

All *SHROOM4* variants identified in this study were predicted to be damaging by one of the silico tools (Supplementary Table 1). Protein sequence alignment indicated that five of six variants are located at residues that are highly conserved across species (Figure 1C). The Arg1430 was less conserved but was predicted to be conserved by GERP (score = 4.15) and phyloP (score = 2.907) (Supplementary Table 2). None of the six affected patients had pathogenic or likely pathogenic variants in genes known to be associated with epileptic phenotypes (Wang et al., 2017).

### Clinical Features

**FIGURE 2:**
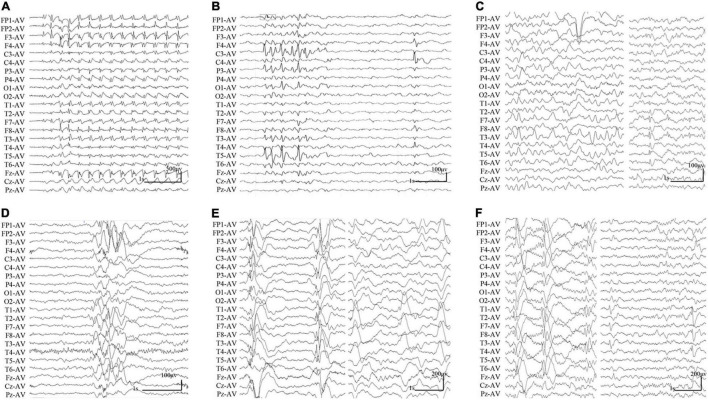
Changes of interictal EEGs and MRI in the cases with *SHROOM4* variants. **(A)** Interictal EEG of case 1 showed 3 Hz generalized spike-slow waves (obtained at the age of 7 years). **(B)** Interictal EEG of case 2 showed bilateral central-temporal independent sharp waves and spikes (obtained at the age of 7 years). **(C)** Interictal EEG of case 3 showed generalized slow waves and left temporal spike-slow waves (obtained at the age of 14 years). **(D)** Interictal EEG of case 4 showed irregular generalized spike-slow waves (obtained at the age of 16 years). **(E)** Interictal EEG of case 5 showed irregular polyspike-slow waves and bilateral temporal independent spike-slow waves (obtained at the age of 4 years). **(F)** Interictal EEG of case 6 showed right predominant generalized spike-slow waves, bilateral temporal independent spike-slow waves (obtained at the age of 3 years).

The clinical features of the six cases with *SHROOM4* variants were summarized in Table 1. The onset age of seizures ranged from 3 years to 16 years old, with a median age of onset of 5.5 years. The patient of case 1 was diagnosed as childhood absence epilepsy (CAE) characterized by absence seizure and 3 Hz generalized spike-slow waves on EEGs (Figure 2A). The patients of case 3, case 4, and case 5 were diagnosed as generalized epilepsy, including Lennox-Gastaut syndrome (LGS), idiopathic generalized epilepsy (IGE), and childhood myoclonic epilepsy (CME); and the three cases had both generalized and focal discharge features on EEGs (Figures 2C−E). The patients of case 2 and case 6 were diagnosed as partial epilepsy, i.e., benign childhood epilepsy with centrotemporal EEG spikes (BECTS) and idiopathic partial epilepsy (IPE), but their EEGs had generalized discharges (asymmetric) (Figures 2B,F). In a word, these patients mainly present with generalized epilepsy, or idiopathic partial epilepsy with generalized seizures or generalized discharge on EEGs. All patients showed normal development. Their brain MRI were normal. These patients all presented good responses to antiepileptic drug and achieved seizure-free.

### Molecular and Molecular Sub-Regional Effects of the *SHROOM4* Variants

**FIGURE 3:**
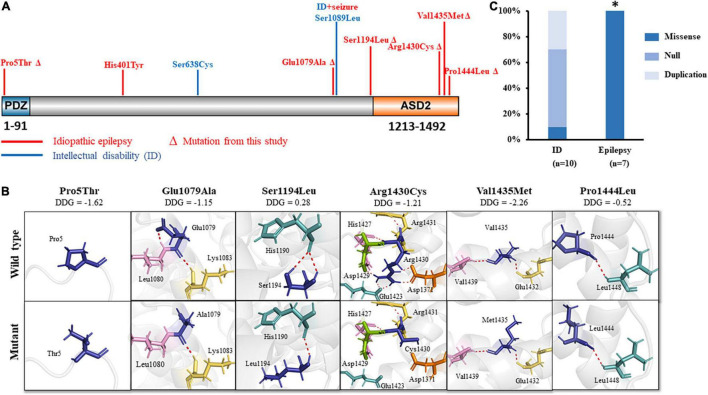
Schematic illustration of *SHROOM4* variants. **(A)** Linear schematic of missense *SHROOM4* mutations and their locations on SHROOM4 protein. Mutations associated with epilepsy were shown in red color. Mutations associated with ID were shown in blue. ΔThe mutation was found in this study **(B)** Changes of hydrogen bonds and free energy change value (DDG) of the mutations. **(C)** The proportion of missense mutations in epilepsy and ID. *The proportion of missense mutations in epilepsy is significantly higher than that in ID.

The SHROOM4 protein contains two evolutionarily conserved domains, i.e., an N-terminal PDZ domain, and a C-terminal ASD2 domain (Hagens et al., 2006; Yoder and Hildebrand, 2007). In the present study, variant Pro5Thr was located in the N-terminal PDZ domain, while Glu1079Ala, Ser1194Leu, Arg1430Cys, Val1435Met, and Pro1444Leu were located in or near to the C-terminal ASD2 domain (Figure 3A).

The molecular effect of the missense variants was analyzed by using I-TASSER for protein modeling and PyMOL 1.7 for visualization (Figure 3B). Three of the variants, including Glu1079Ala, Ser1194Leu, and Arg1430Cys, changed hydrogen bonds with the surrounding residues. Originally, Glu1079 formed two hydrogen bonds with residues Leu1080 and Lys1083, respectively. When glutamine was replaced by alanine, the hydrogen bonds with Leu1080 was destroyed. Ser1194 formed two hydrogen bonds with His1190. When serine was replaced by leucine, one of the hydrogen bond was destroyed. Arg1430 formed seven hydrogen bonds with residues Asp1371, Glu1423, His1427, Asp1429, and Arg1431, respectively. When arginine was replaced by cystine, five hydrogen bonds with Asp1371, Glu1423, and Asp1429 were destroyed, and only the hydrogen bonds with His1427 and Arg1431 were kept.

The other three variants, including Pro5Thr, Pro1444Leu, and Val1435Met, have no changed in hydrogen bonds, but they were predicted to decrease the protein stability significantly (ΔΔG values < −0.5 kcal/mol) (Figure 3B).

### Genotype-Phenotype Correlation

Previously, 12 *SHROOM4* variants have been reported, including 3 missense (Hagens et al., 2006; Farwell et al., 2015; Routier et al., 2019), 2 duplications (Froyen et al., 2007; Isrie et al., 2012), and 7 destructive variants (1 nonsense, 3 gross deletion variants (Honda et al., 2010; Armanet et al., 2015; Danyel et al., 2019; Heide et al., 2020), and 3 complex rearrangements (Hagens et al., 2006; Dong et al., 2021)) (Supplementary Table 2). Eleven of the variants associated with intellectual disability (ID), of which 9 were destructive mutations or duplications (Figure 3C). Among the three missense variants, one missense variant was associated with ID (Farwell et al., 2015) and one missense variant was associated with ID and seizures (Hagens et al., 2006). Additional one missense variant (His401Tyr) was reported to be associated with myoclonic-atonic epilepsy (MAE) (Routier et al., 2019). These variants are all hemizygous. In this study, all hemizygous variants were missense variants, which were associated with idiopathic epilepsy without ID (Figure 3A). The proportion of missense variants in epilepsy (7/7) was significantly higher than that in ID (1/10) (p < 0.001) (Figure 3C), suggesting a genotype-phenotype correlation.

## Discussion

*SHROOM4* gene encodes an actin-binding proteins, which plays an important role in cytoskeletal architecture, synaptogenesis, and maintaining GABA_*B*_ receptor-mediated inhibition. Hemizygous missense variants were identified in six cases with idiopathic epilepsy without intellectual disability (Figure 1A). All patients presented with features of generalized seizures or generalized discharges. These hemizygous variants had no or extremely low allele frequencies in controls and showed statistically higher frequency in the case cohort than controls (Table 2). All variants were predicted to alter hydrogen bond with surrounding amino acids or decreased protein stability. This study suggests that *SHROOM4* is potentially a candidate causative gene of X-link epilepsy with features of generalized seizures or generalized discharges.

The *SHROOM4* gene is widely expressed in the brain^2^. It plays a critical role in regulating dendritic spine morphology and controls the cell surface expression and intracellular trafficking of GABA_*B*_ receptor (Zapata et al., 2017), which inhibit neuronal activity through G protein-coupled second-messenger systems (Euro, 2014; Yoo et al., 2017). In rat, *Shrm4* were found to influence hippocampal excitability by modulating tonic inhibition in dentate gyrus granule cells. Knockdown of *Shrm4* cause increased susceptibility to seizures (Zapata et al., 2017). Previously, a study identified a hemizygous missense variant in a family with ID and seizures (Hagens et al., 2006). Recently, a missense variant with unknown origin was also found in a male with MAE (Routier et al., 2019). However, the relationship between *SHROOM4* mutations and epilepsy remains uncertain. In this study, we identified six novel hemizygous missense variants in six unrelated cases with idiopathic epilepsy but without ID, suggesting that *SHROOM4* is potentially a candidate gene of epilepsy.

Previous studies have shown that mutations of the GABA receptors, such as, *GABRA1*, *GABRA5*, *GABRA6, GABRB3, GABRD*, *GABRG2*, were associated with idiopathic generalized epilepsy (Cossette et al., 2002; Hirose, 2006; Moller et al., 2017; Lee et al., 2018; May et al., 2018). Mutations of the GABA_*B*_ receptor cause generalized epilepsy by impairing inhibitory network neurodevelopment (Samarut et al., 2018). Recent study found that knockdown of *Shrm4* severely impairs GABA_*B*_ receptor-mediated inhibition and thus potentially associated with generalized epilepsy. In the present study, patients with *SHROOM4* variants presented mainly generalized epilepsy, such as CAE, MAE, LGS, IGE, and those with partial seizures also had bilateral or generalized discharge or generalized seizures (Table 1 and Figure 1), potentially suggesting an association between *SHROOM4* variants and generalized epilepsy. The patients with *SHROOM4* mutations showed good responses to proper antiepileptic treatment and got seizure free, in spite of frequent daily seizures in several cases. These findings suggested that the establishment of *SHROOM4*-epilepsy association would be potentially significant in management of the patients with *SHROOM4* mutations.

Previously, 11 variants were reported in patients with ID (Ng et al., 2004), 9 of which were destructive mutations or duplications (Figure 3C and Supplementary Table 2). Only two of those variants were missense variants. In contrast, epilepsy-associated variants were all missense variants (Figure 3C), suggesting a genotype-phenotype correlation.

*SHROOM4* gene encodes a member of the Shroom family, which contains an N-terminal PDZ domain and a C-terminally positioned motif termed ASD2 (Hagens et al., 2006; Yoder and Hildebrand, 2007). The PDZ domain interacts with C terminus of GABA_*B*_ receptors, while the ASD2 domain is capable of inducing myosin II-dependant changes in cell shape. The central portion of the protein appears to be an actin targeting sequence and mediated Shrm4 localization (Yoder and Hildebrand, 2007). The current data demonstrated that two missense variants located in or closed to N-terminal PDZ domain were associated with generalized epilepsy; two missense variants located in the middle of protein were associated with ID, one of which was located near to C-terminal ASD2 domain and associated with epilepsy (Hagens et al., 2006; Farwell et al., 2015); missense variants located in or near to C-terminal ASD2 domain were associated with epilepsy with both focal and generalized seizures or discharges. These evidences suggested a possible molecular sub-regional effect of *SHROOM4* variants, as that in several genes reported previously (Wang et al., 2018; Liu et al., 2020; Tang et al., 2020). However, further studies are required to determine the function details of each region and their association with different phenotypes.

This study has several limitations. Knockdown, i.e., LOF of Shrm4, in rat resulted in severely impaired synaptogenesis and reduced GABA_*B*_ receptor-mediated inhibition, causing susceptibility to seizures. The probability of being LOF intolerant (pLI) score was high for *SHROOM4* (pLI = 0.997), suggesting that *SHROOM4* is intolerant to LOF variants. However, the specific mechanism of epileptogenesis of the *SHROOM4* variants remains unknown; and further experimental studies are required to determine the functional consequence of the variants. Additionally, although these missense variants were associated with generalized epilepsy, the patients presented different epilepsy syndromes. The mechanism of phenotype variation warrants further studies.

In conclusion, we identified six novel *SHROOM4* hemizygous missense variants in epilepsy patients with features of generalized seizures or generalized discharges. Further analysis revealed that a potential genotype-phenotype correlation and sub-regional molecular implication of *SHROOM4* variants. This study potentially extends the spectrum of diseases phenotype associated with *SHROOM4* variants and helps to understand the mechanisms of phenotypic variation.

## Data Availability Statement

The original contributions presented in the study are included in the article/Supplementary Material, further inquiries can be directed to the corresponding author/s.

## Ethics Statement

The studies involving human participants were reviewed and approved by the Ethics Committee of The Second Affiliated Hospital of Guangzhou Medical University. Written informed consent to participate in this study was provided by the participants’ legal guardian/next of kin.

## Author Contributions

Y-HY designed the study, administered the project, and revised the manuscript. W-JB completed collection of the clinical data, analyzed the data, and draft of the manuscript. Z-JL analyzed the data and drafted of the manuscript. SL, NH, L-DG, B-ML, and JW contributed to data analysis and interpretation. B-ML and L-DG performed data analysis and provided technical assistance. All authors have read and approved the final manuscript.

## Conflict of Interest

The authors declare that the research was conducted in the absence of any commercial or financial relationships that could be construed as a potential conflict of interest.

## Publisher’s Note

All claims expressed in this article are solely those of the authors and do not necessarily represent those of their affiliated organizations, or those of the publisher, the editors and the reviewers. Any product that may be evaluated in this article, or claim that may be made by its manufacturer, is not guaranteed or endorsed by the publisher.

## Footnotes

1
http://varcards.biols.ac.cn/

2
https://www.gtexportal.org/home/gene/SHROOM4
