# Supplementary material for: SHROOM4 Variants Are Associated With X-Linked Epilepsy With Features of Generalized Seizures or Generalized Discharges
Source: Front Mol Neurosci. 2022 May 17;15:862480. doi: 10.3389/fnmol.2022.862480 (PMC9157246; doi:10.3389/fnmol.2022.862480)
Supplement: Supplementary file 1 [file Table_1.DOCX]

| Case | Coordinate | cDNA change (NM_020717) | Protein change | Inheritance | PP2 | SIFT | FATHMM_MKL | M_CAP | CADD | GenoCanyon | GERP_pred | phyloP_pred |
| --- | --- | --- | --- | --- | --- | --- | --- | --- | --- | --- | --- | --- |
| Case 1 | chrX:50557006 | c.13C>A | p.Pro5Thr | maternal | P (0.969) | D (0.002) | D (0.829) | T (0.013) | T (12.29) | D (1) | C (3.18) | C (3.105) |
| Case 2 | chrX:50350906 | c.3236A>C | p.Glu1079Ala | maternal | P (0.989) | D (0.003) | D (0.903) | D (0.114) | D (18.32) | D (1) | C (5.65) | C (4.429) |
| Case 3 | chrX:50350561 | c.3581C>T | p.Ser1194Leu | maternal | P (0.553) | T (0.086) | T (0.048) | T (0.004) | T (11.35) | T (0.986) | C (3.3) | C (2.218) |
| Case 4 | chrX:50339889 | c.4288C>T | p.Arg1430Cys | maternal | P (1) | D (0) | D (0.956) | D (0.64) | D (29.4) | D (1) | C (4.15) | C (2.907) |
| Case 5 | chrX:50339874 | c.4303G>A | p.Val1435Met | maternal | P (0.999) | D (0.006) | D (0.984) | D (0.63) | D (29.3) | D (1) | C (5.96) | C (7.483) |
| Case 6 | chrX:50339846 | c.4331C>T | p.Pro1444Leu | maternal | B (0.137) | T (0.171) | T (0.429) | D (0.337) | D (23.4) | T (0.998) | C (3.77) | NC (1.635) |

**Supplementary Table 1 Genetic Feature of the Individuals with** ***SHROOM4* variants**

Abbreviations are as follows: B, benign; C, conserved; CADD, Combined Annotation Dependent Depletion; D, damaging; NC, nonconserved; P, probably_damaging; PP2, Polyphen2_HDIV; T, tolerable;

***SHROOM4***: RefSeq transcript NM_020717.5
