# Supplementary material for: SHROOM4 Variants Are Associated With X-Linked Epilepsy With Features of Generalized Seizures or Generalized Discharges
Source: Front Mol Neurosci. 2022 May 17;15:862480. doi: 10.3389/fnmol.2022.862480 (PMC9157246; doi:10.3389/fnmol.2022.862480)
Supplement: Supplementary file 2 [file Table_2.DOCX]

**Table S2. Summary of Clinical Features in the Cases with *SHROOM4* Mutations**

| **Mutation type** | **Mutation** | **Phenotype** | **Inheritance** | **Reference** |
| --- | --- | --- | --- | --- |
| missense | c.1201C>T/p.H401Y | Myoclonic atonic epilepsy | NA | Routier (2019) |
|  | c.1913C>G/p.S638C | Mental retardation, Stocco dos Santos type | maternal | Farwell (2015) |
|  | c.3266C>T/p.S1089L | Mental retardation, X-linked | maternal | Hagens (2006) |
| nonsense | c. 2050C>T/p.R684* | Mental retardation, Stocco dos Santos type | NA | Heide (2020) |
| Gross deletions | ~2.67 mb | Mental retardation, X-linked | NA | Honda (2010) |
|  | 2.6 Mb incl. entire gene & 22 others | Psychomotor retardation | maternal | Armanet (2015) |
|  | 700 kb incl. ex. 6-9 + entire CLCN5 and 3 other genes | Intellectual disability, short stature, and microcephaly | maternal | Danyel (2019) |
| Gross insertions | 0.26Mb incl part of gene & DGKK | Developmental delay, choanal atresia, ventricle septum defect & camptodactyly | NA | Isrie (2012) |
|  | 3.1 Mb, entire gene | Mental retardation, X-linked | De novo | Froyen (2007) |
| Complex rearrangements | 4.6 Mb dupl. and insertion dup(X)(p11.22p11.21) | Developmental delay and autism spectrum disorder | NA | Dong (2021) |
|  | Balanced translocation 46,X,t(X;19)(p11.2;p13.3) | Developmental delay and autism spectrum disorder | De novo | Hagens (2006) |
|  | Balanced translocation 46,X,t(X;8)(p11.2;p22.3) | Mental retardation, X-linked | De novo | Hagens (2006) |
